# Circulating Levels of the Cardiovascular Biomarkers ST2 and Adrenomedullin Predict Outcome within a Randomized Phase III Lung Cancer Trial (RASTEN)

**DOI:** 10.3390/cancers14051307

**Published:** 2022-03-03

**Authors:** Emelie Gezelius, Pär-Ola Bendahl, Widet Gallo, Kelin Gonçalves de Oliveira, Lars Ek, Bengt Bergman, Jan Sundberg, Olle Melander, Mattias Belting

**Affiliations:** 1 Department of Clinical Sciences, Lund, Division of Oncology, Lund University, Barngatan 4, SE-221 85 Lund, Sweden; par-ola.bendahl@med.lu.se (P.-O.B.); kelin.goncalves_de_oliveira@med.lu.se (K.G.d.O.); mattias.belting@med.lu.se (M.B.); 2 Department of Respiratory Medicine, Lund University Hospital, Entrégatan 7, SE-221 85 Lund, Sweden; l.ek@icloud.com; 3 Clinical Research Centre, Hypertension and Cardiovascular Disease Group, Department of Clinical Sciences, Skåne University Hospital, Lund University, Jan Waldenstroms gata 35, SE-214 28 Malmo, Sweden; widet.gallo@med.lu.se (W.G.); olle.melander@med.lu.se (O.M.); 4 Department of Respiratory Medicine, Sahlgrenska University Hospital, SE-413 45 Gothenburg, Sweden; bengt.bergman@lungall.gu.se; 5 Department of Hematology, Radiophysics and Oncology, Skåne University Hospital, Lasarettsgatan 23A, SE-221 85 Lund, Sweden; jan.sundberg@skane.se; 6 Department of Immunology, Pathology, and Genetics, Uppsala University, Rudbecklaboratoriet, SE-751 85 Uppsala, Sweden

**Keywords:** small cell lung cancer, cardiovascular biomarkers, individualized treatment

## Abstract

**Simple Summary:**

Cardiovascular disease is common in patients with small cell lung cancer, partly reflecting its high correlation with smoking. Cardiovascular comorbidities may limit patient tolerance to cytotoxic drugs, thereby influencing the choice and intensity of treatment and, ultimately, patient survival. In light of the challenges relating to assessing cardiovascular status clinically in newly diagnosed lung cancer, objective biomarkers of cardiovascular vulnerability are warranted. Here, we show that circulating levels of ST2, an established biomarker in heart failure, and adrenomedullin, a vasodilator peptide known to reflect several aspects of cardiovascular status, strongly correlate with survival in small cell lung cancer. Our data, which are based on a large, randomized trial cohort, suggest the potential use of cardiovascular biomarkers in guiding clinicians in making individualized treatment decisions.

**Abstract:**

Cardiovascular comorbidity is common in small cell lung cancer (SCLC) and may significantly affect treatment tolerability and patient outcome. Still, there are no established biomarkers for objective and dynamic assessment as a tool for improved treatment decisions. We have investigated circulating levels of midregional-pro-adrenomedullin (MR-proADM), midregional-pro-atrial-natriuretic peptide (MR-proANP), copeptin (surrogate for vasopressin) and suppression-of-tumorigenicity-2 (ST2), all known to correlate with various aspects of cardiovascular function, in a SCLC cohort (*N* = 252) from a randomized, controlled trial (RASTEN). For all measured biomarkers, protein levels were inversely associated with survival, particularly with ST2 and MR-proADM, where the top versus bottom quartile was associated with an adjusted hazard ratio of 2.40 (95% CI 1.44–3.98; *p* = 0.001) and 2.18 (95% CI 1.35–3.51; *p* = 0.001), respectively, in the entire cohort, and 3.43 (95% CI 1.73–6.79; *p* < 0.001) and 3.49 (95% CI 1.84–6.60; *p* < 0.001), respectively, in extensive disease patients. A high combined score of MR-proADM and ST2 was associated with a significantly reduced median OS of 7.0 months vs. 14.9 months for patients with a low combined score. We conclude that the cardiovascular biomarkers MR-proADM and ST2 strongly correlate with survival in SCLC, warranting prospective studies on the clinical utility of MR-proADM and ST2 for improved, individualized treatment decisions.

## 1. Introduction

Lung cancer is the worldwide leading cause of death from cancer [1]. Small cell lung cancer (SCLC), an aggressive subtype accounting for ~15% of all lung cancer cases [2], is associated with a particularly poor prognosis, with 5-year survival rates of ~5% [3,4]. Despite recent therapeutic advances, including the emergence of immunotherapy in the care of SCLC, the overall effects are yet to be seen. Comorbidity is common in patients with SCLC and the trend is increasing. The presence of one or more comorbidity was reported in 76% of SCLC patients in 2011–2012, compared to 55% in 1995–1998 [5], with cardiovascular (CV) and pulmonary disease being among the most prevalent. In this large cohort (*N* = 4142), CV disease (CVD) was observed in 36% and 24% in male and female patients, respectively [5]. This is consistent with other studies, reporting CVD in 42%–48% of SCLC patients [6,7]. 

CV comorbidity in cancer patients may limit chemotherapy tolerability and patient outcome [8]. In lung cancer, the association between comorbidity and survival remains unclear. Some evidence indicates that a Simplified Comorbidity Score, developed by Colinet et al. [9], of ≥9 correlates with poor outcome [7], whereas others have suggested that CV comorbidity rather has an indirect effect on survival through predicting treatment choice [5,6]. However, it is important to consider that CVD encompasses a wide spectrum of conditions, with varying degrees of clinical manifestation, and its systemic impact during anti-tumoral treatment may be challenging to assess. In fact, current CV status and vulnerability may be difficult to monitor, particularly in the light of an aggressive malignancy, and the additional CV stress imposed by toxic treatment. Together, this poses a significant clinical challenge. Hence, biomarkers that can objectively and dynamically monitor CV status are called for as an additional tool for guidance in individualized treatment decisions in SCLC. 

Notably, whereas cancer patients undergoing systemic treatment are routinely monitored for renal, hepatic and bone marrow function using established laboratory parameters, biomarkers that may reflect CV status remain unexplored and are rarely assessed. Among the interesting candidates, suppression of tumorigenicity 2 (ST2, also called IL-1 receptor-like 1) and the vasoactive peptides adrenomedullin (ADM), atrial natriuretic peptide (ANP), and arginine vasopressin (AVP) have been described to reflect various aspects of CV pathology [10]. Soluble ST2 isoform (sST2), which is measurable in plasma, predicts mortality and morbidity in several CV conditions, and is now an FDA-approved biomarker in heart failure [10,11]. The functional ligand IL-33, that upon binding to membrane-bound ST2 (ST2L) promotes an inflammatory response mainly through Th2 effector cells, has been shown to exert a cardioprotective effect [12,13]. On the contrary, sST2 may act as a decoy receptor and thus attenuate the inflammatory and cardioprotective effects of IL-33. ADM, ANP and AVP, commonly defined as vasoactive peptides, are involved in maintaining CV homeostasis and electrolyte balance [14,15,16]. ADM and AVP both reflect neurohumoral activation, a hallmark of heart failure, whereas ANP is a biomarker of myocardial stretching, closely resembling brain natriuretic peptide, a well-established marker of congestive heart failure. All three peptides correlate independently with mortality in heart failure [10]. Due to their short half-lives in plasma, measurement of the stable peptide precursors midregional pro-ADM (MR-proADM), midregional pro-ANP (MR-proANP) and C-terminal pre-provasopressin (copeptin) is preferred [17,18,19]. 

In the present study, we were interested in elucidating whether circulating levels of candidate biomarkers of CV stress may predict the outcome of SCLC patients. In a clinically well-annotated cohort of SCLC patients within a randomized controlled trial [20], we demonstrate that high levels of MR-proADM and ST2 strongly correlate with worse patient outcome, suggesting their potential use in clinical risk stratification and monitoring of SCLC. 

## 2. Materials and Methods

### 2.1. RASTEN Clinical Trial

A full description of the clinical trial design has previously been reported [20]. In brief, RASTEN is an international, randomized phase-3 trial of standard treatment with or without the addition of the low-molecular-weight heparin enoxaparin in SCLC (ClinicalTrials.gov: NCT00717938), with the primary aim to test for improved survival. Standard therapy in both arms included a platinum compound and topoisomerase inhibitor, and radiotherapy was given according to local protocol. In the intervention arm, enoxaparin was administered at 1 mg/kg as daily subcutaneous injections for the duration of the chemotherapy regimen. The study was carried out in agreement with the Declaration of Helsinki with approval from the Regional Ethics Committee at Lund University, Sweden.

### 2.2. Patient Selection and Plasma Sampling

Blood samples were collected continuously during the clinical trial, and for the present sub-study baseline samples (prior to start of chemotherapy) were used. Plasma was collected in EDTA-tubes and stored in a −80 °C freezer at the Clinical Research Unit, Skåne University Hospital, Lund, Sweden. The present biomarker cohort was established at the cut-off date of 1 November 2013, consisting of the first consecutive 292 patients.

### 2.3. Clinical Outcome

The primary endpoint, overall survival (OS), was defined as the date of randomization to the date of death from any cause. For patients not reported dead, information regarding vital status was confirmed from each study center before data collection cut-off on 4 April 2017. Progression-free survival (PFS) was measured from the date of randomization to the date of objective or clinical progression or death from any cause, whichever came first.

### 2.4. Immunoassay Analysis of Vasoactive Peptides

Absolute levels of stable fragments of the peptide precursors MR-proADM, MR-proANP and copeptin were measured in EDTA-plasma using a standardized, commercial immunoluminometric sandwich assay, as previously described (KRYPTOR, Thermo Fisher Scientific, Hennigsdorf/Berlin, Germany) [17,18,19]. 

### 2.5. Proximity Extension Assay

Total ADM and ST2 were determined in EDTA-plasma at baseline using the Proseek Multiplex Oncology 1-v2^96×96^ and CVD 1^96×96^ panels (Olink Bioscience, Uppsala, Sweden), as previously described [21]. The proximity extension assay (PEA) provides high sensitivity and specificity based on oligonucleotide-labelled antibody probe pairs binding to their specific target protein, generating a PCR-amplified DNA template, which is proportional to the initial antigen concentration as quantified by real-time qPCR. The values are reported as normalized protein expression (NPX) in arbitrary units. Four internal and three negative controls were used to calculate the lower limit of detection (LOD) for each protein. All assays were performed by collaborators blinded to the study endpoint.

### 2.6. Statistical Analysis 

The statistics packages SPSS v27 and STATA v16 were used for statistical analysis. Biomarkers were categorized into quartiles to allow for non-linear effects on survival. Survival was estimated using the Kaplan–Meier method, and evidence for difference in survival between groups of patients was evaluated using the log rank test. Cox regression was used to calculate hazard ratios (HRs). Proportional hazards assumptions were checked graphically, and deviations were handled by restricting the follow-up to 12 months in some analyses. Stepwise backward logistic regression, with survival at 12 months yes/no as the outcome, was used to find a set of variables with jointly strong association to survival. Due to missing data for some of the candidate variables, 10 complete datasets were constructed using multiple imputation with chained equations. Linear regression imputation models were used for all variables with missing data, and the complete set of predictors evaluated in the study were used as predictors in these models. Missingness should be non-random and conditional on the other predictors and, hence, the missing at random assumption was reasonably well met. All the three possible dichotomizations of the quartiles of the experimental biomarkers were evaluated in the stepwise modelling procedure. Three variables were highly significant (*p* < 0.01) in the final averaged multiple imputation model as well as in each of the 10 individual imputed datasets: MR-proADM, ST2 and tumor stage. Note the exploratory nature of this modelling procedure. A combined biomarker score, ranging between 2 and 8 points, was generated by adding the scores 1 to 4 representing the quartiles of MR-proADM and ST2. A high combined score was defined as 6–8 points and a low combined score as 2–5 points. Multivariable Cox models were used to calculate HRs adjusted for the well-recognized prognostic factors age, gender, performance status (WHO 0–1 vs. 2–3) and disease stage (limited vs. extensive disease). Leukocytosis (leukocyte count ≤9.0 vs. >9 × 10^9^/L) and hyponatremia (plasma sodium levels <136 vs. ≥136 mmol/L) were also included in the multivariate models as these variables were found to correlate with survival in univariate analysis, which is consistent with the existing literature [22,23]. Spearman rank correlation was used for correlations between different assays.

## 3. Results

### 3.1. Study Population

MR-proADM, MR-proANP, copeptin, and ST2 were determined at baseline in treatment-naïve SCLC patients to exclude potential interaction effects of prior cytotoxic therapy. Of the first 292 consecutively enrolled patients in the RASTEN trial, 40 patients were excluded from the biochemical analysis due to poor sample quality (*N* = 11), unavailable sample (*N* = 15) or inclusion criteria not being fulfilled (*N* = 14) (Supplementary Figure S1). A total of 252 patients were included in the present cohort, of which 104 (41%) had limited disease (LD) and 148 (59%) had extensive disease (ED) at presentation. Baseline demographics are displayed in Table 1. 

### 3.2. Cardiovascular Biomarkers at Baseline

Using a standardized, immunoluminometric assay, MR-proADM was measured in all 252 samples, whereas MR-proANP and copeptin were analyzed in 251 and 194 samples, respectively. Using the proximity extension assay, ADM and ST2 were determined in 242 samples. Baseline levels of the peptides are shown in Supplementary Table S1. For MR-proADM, MR-proANP and copeptin, the median protein concentrations were 0.76 nmol/L, 75.6 pmol/L and 7.7 pmol/L, respectively. In comparison, the concentrations of MR-proADM, MR-proANP and copeptin were previously reported as 0.75 nmol/L, 125.5 pmol/L and 9.6 pmol/L, respectively, in an age-matched, prospective, population-based cohort (*N* = 5415) [24].

### 3.3. Cardiovascular Biomarkers and Clinical Outcome 

In unadjusted Cox regression analyses, all biomarkers correlated with survival when comparing the top vs. bottom quartiles, and the effect was most prominent in patients with ED (Table 2). Strong prognostic evidence was seen for all of the biomarkers except MR-proANP, when adjusting for tumor stage, performance status, age, gender, leukocytosis and hyponatremia (Table 3). 

**Figure 1:**
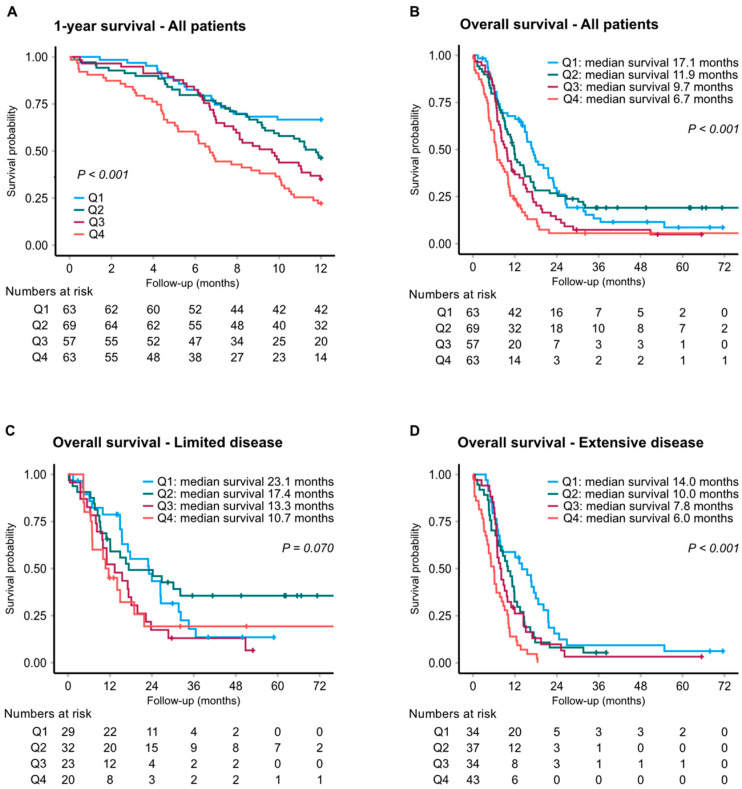
Kaplan–Meier analysis of survival by MR-proADM levels. One-year survival (**A**) and overall survival for all patients (**B**) and by stage; limited (**C**) and extensive (**D**) disease.

**Figure 2:**
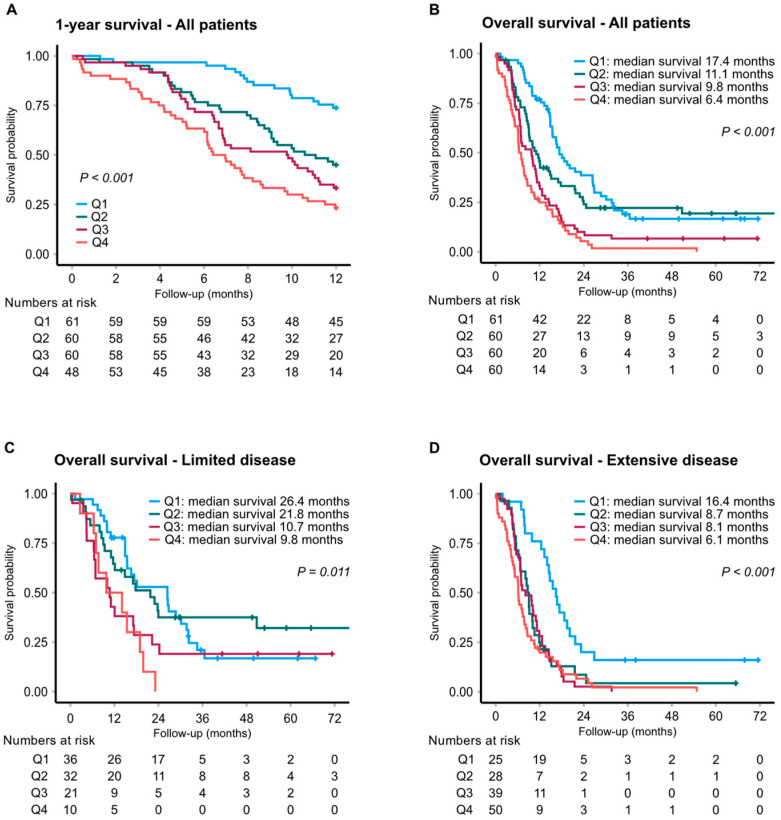
Kaplan–Meier analysis of survival by ST2 levels. One-year survival (**A**) and overall survival for all patients (**B**) and by stage; limited (**C**) and extensive (**D**) disease.

Based on MR-proADM, the median overall survival (OS) was 6.7 and 17.1 months in patients with the highest and lowest MR-proADM levels, respectively (adjusted HR: 2.18; 95% CI 1.35–3.51; *p* = 0.001) (Figure 1). Subgroup analysis by disease extent gave an adjusted HR for overall survival of 3.49 (95% CI 1.84–6.60; *p* < 0.001) in patients with ED (Figure 1D). To corroborate these findings, ADM was further assessed by a commercially available proximity extension assay (OLINK), showing a strong correlation with the MR-proADM assay (Spearman’s coefficient of 0.879) (Supplementary Figure S2). Accordingly, OLINK-ADM levels showed a comparable association with patient OS (Table 2 and Table 3, and Supplementary Figure S3). For ST2, median OS was 6.4 and 17.4 months when comparing the top versus bottom quartiles, corresponding to an adjusted HR of 2.40 (95% CI 1.44–3.98; *p* = 0.001) for the entire cohort (Figure 2), and 3.43 (95% CI 1.73–6.79; *p* < 0.001) in ED (Figure 2D). Survival graphs by distribution of MR-proANP and copeptin are illustrated in Supplementary Figures S4 and S5.

### 3.4. Prediction Models and Combined Biomarker Score

**Figure 3:**
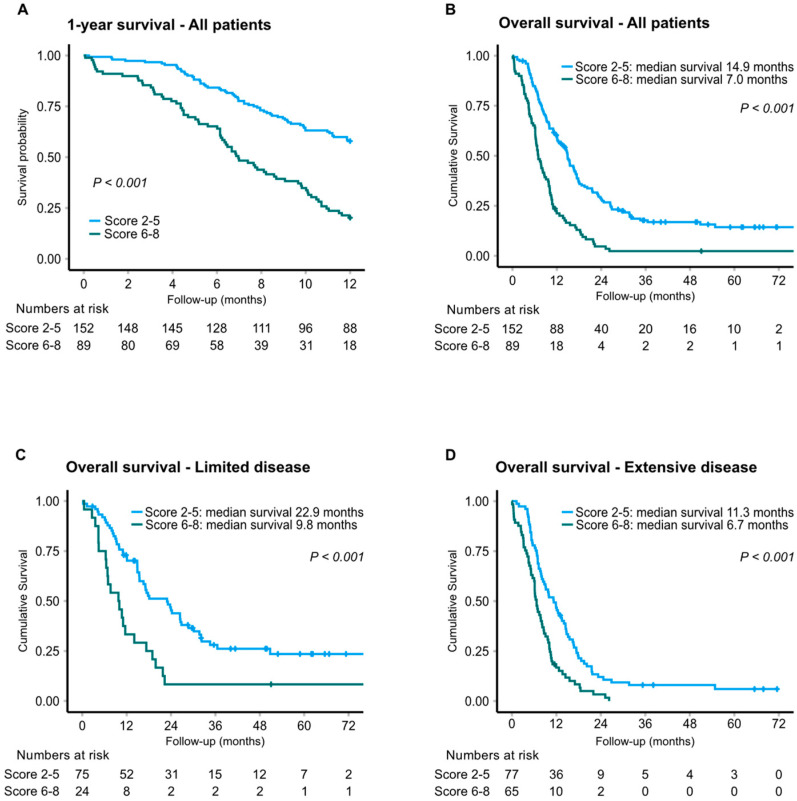
Kaplan–Meier analysis of survival by combined biomarker score of MR-proADM and ST2. One-year survival (**A**) and overall survival for all patients (**B**) and by stage; limited (**C**) and extensive (**D**) disease.

Prediction modelling incorporating the CV biomarkers as well as clinical predictive factors and routine laboratory parameters identified MR-proADM, ST2, and tumor stage as the main prognostic factors with respect to 1-year survival. Hence, MR-proADM and ST2 were further considered for a combined biomarker score. Combining the quartile scores for ST2 and MR-proADM revealed that a high score of 6–8 was associated with a significantly reduced median OS of 7.0 months, compared to 14.9 months for patients in the lower quartiles (Figure 3). In ED patients, median OS was 11.3 and 6.7 months in patients with a combined score of 2–5 versus 6–8, respectively (unadjusted HR: 2.04; 95% CI 1.44–2.89; *p* < 0.001). Notably, in LD, a low quartile score corresponded to a median OS of 22.9 months (*N* = 75) compared to 9.8 months in patients with a high score (*N* = 24) (unadjusted HR: 2.43; 95% CI 1.46–4.04; *p* = 0.001). Accordingly, at two years of follow-up, 41% were alive in the LD low-score subgroup, compared to 8% in the LD high-score subgroup. Estimations of PFS based on MR-proADM, ST2 and combined score showed strong correlations between each of the biomarkers and PFS, particularly for ST2 (*p* < 0.001). For patients with a high vs. low combined score, median PFS was 5.2 months and 8.1 months, respectively (*p* < 0.001) (Supplementary Figure S6).

## 4. Discussion

Although cardio-oncology has received increasing attention in the management of cancer patients, there are still no established lab assays that link CV biomarkers to patient outcome. Here, we show that baseline levels of ADM and ST2 are significantly and independently associated with SCLC-patient survival in a dose-dependent manner. Subgroup analysis by tumor stage identified LD patients with a low ADM-ST2 biomarker score to have a particularly favorable prognosis, showing a median OS of 22.9 months. Moreover, LD patients with a high score had a shorter survival than ED patients with a low score (median OS of 9.8 and 11.3 months, respectively), suggesting that ADM and ST2 add prognostic value to established, clinical parameters. This may have potential clinical utility, i.e., low-scoring patients, regardless of tumor stage, should be managed ambitiously with chemo- and radiotherapy, and potentially in combination with immune checkpoint inhibitors. On the contrary, patients with a high biomarker score may be considered for more conservative, dose-limiting treatment, and dynamically monitored by ADM-ST2 biomarkers to assess potential CV toxicity. In addition, the combined score correlated significantly to PFS, further supporting its prognostic value. This may also suggest that tolerance to chemotherapy and, hence, response to treatment, was reduced in patients with CV comorbidity, but this interpretation must be viewed with caution and addressed in follow-up studies. Another aspect of our findings is the potential use of biomarkers to identify SCLC patients with subclinical CVD who may benefit from interventions aimed at optimizing CV function prior to or during anti-tumoral treatment. The incorporation of treatment with enoxaparin, i.e., the study arm of the RASTEN trial, as an interaction term in multivariable regression models did not yield any differential treatment effect based on biomarker levels (*p* = 0.44 and 0.29 for ADM and ST2, respectively). Hence, biomarker levels did not predict response to low-molecular-weight heparin.

It should be noted that ST2 may correlate with systemic conditions other than CV stress. In addition to its implications in CVD, sST2 has been shown to correlate with short and long-term survival in acute exacerbations of chronic obstructive pulmonary disease (COPD) [25]. The anti-ST2 antibody MSTT1041A (astegolimab; Genentech) is currently being explored in several phase II trials, including in COPD (NCT03615040), uncontrolled asthma (NCT02918019) and severe coronavirus disease 2019 pneumonia (NCT04386616), highlighting its importance also in respiratory pathology. Despite its name, evidence is conflicting regarding the role of ST2 in tumor disease. Interestingly, Jeught et al. [26] suggested an active role of ST2 in the immunosuppressive tumor microenvironment of colorectal cancer (CRC), and elevated ST2 was found to be associated with poor survival and deficient CD8+ T-cell cytotoxicity. Combined targeting of programmed cell death protein 1 (PD-1) and ST2 significantly reduced tumor growth in mice, pointing to ST2 as a potential target for immunotherapy. However, another study [27] demonstrated an inverse relationship between sST2 levels and CRC tumor growth, implying a tumor-inhibiting effect of sST2. In breast cancer, increased levels of serum sST2 were found in patients with estrogen receptor-positive tumors compared to healthy controls. This correlated with known prognostic factors, but not with survival [28]. To the best of our knowledge, ST2 has not previously been investigated in SCLC. 

In an unselected cancer population, including non-SCLC patients (*N* = 61), NT-proBNP, MR-proANP, MR-proADM and copeptin were increased as compared with healthy controls, and related to pro-inflammatory cytokines and long-term mortality [29]. Apart from being a CV biomarker, substantial evidence indicates that ADM is actively involved in malignant processes, such as angiogenesis [30,31,32,33]. ADM is strongly induced by hypoxia, and overexpression of ADM has been reported in several malignant conditions, including pancreatic, colorectal, and renal cancer [30,34,35,36,37]. Plasma ADM has not previously been linked to disease progression in SCLC, and immunohistochemical studies found no or weak expression of ADM in SCLC tissue [38,39]. However, in line with our results, plasma ADM has been correlated with tumor progression in neuroendocrine carcinomas of various origin [40]. With regard to copeptin, a surrogate marker for AVP, our findings are consistent with Umemura et al. [41], investigating mature serum AVP in 34 patients with SCLC where increased levels were associated with reduced survival. It is unclear whether AVP is actively involved in carcinogenesis, although an indirect effect on angiogenesis by stimulated secretion of VEGF and endothelin-1 was suggested in vitro [42,43]. However, the synthetic AVP analogue [V^4^Q^5^] dDAVP showed inhibitory effects on metastasis, tumor growth and angiogenesis in animal models of CRC [44]. Together, our and previous results motivate future mechanistic studies that unravel the expression and functional role of especially ADM and ST2 in SCLC and other cancers to understand how these peptides may locally influence the tumor microenvironment and represent tractable targets for anti-tumoral therapies.

Our study has some limitations, and based on the present findings, a follow-up, prospective trial that includes, e.g., cardiac ultrasound should investigate whether the increased mortality in patients with high biomarker levels solely can be explained by CV failure, acknowledging that these markers may be raised in other systemic stress conditions. On the other hand, based on the inclusion and exclusion criteria of the RASTEN trial, it is unlikely that patients affected by overt, systemic illness were enrolled. Notably, baseline levels of MR-proADM were comparable to an age-matched, large, population-based cohort [24], indicating that our results are not affected by a disproportional prevalence of manifest CVD prior to start of chemotherapy. Secondly, smoking is a major risk factor in CVD as well as in SCLC; indeed, less than 2% of SCLC patients are never-smokers [45]. An early study did not demonstrate any effects on survival in SCLC patients who continued smoking during treatment compared to patients who stopped smoking [46], whereas others have reported improved prognosis with smoking cessation specifically in LD patients [47,48]. Experimental models have suggested that aryl hydrocarbons can upregulate ADM [49] and affect the expression of ST2 [50], therefore representing a potential confounder. Meanwhile, the proximity ligation assay (OLINK) used for ST2 measurement does not provide absolute concentrations, which makes it difficult to directly compare with data obtained by ELISA or the FDA-approved assay (Presage^®^ ST2 Assay). However, assessment of ST2 using the OLINK platform and a commercially available ELISA has previously shown excellent correlation [25]. Moreover, in the present study, ADM determined by the proximity extension assay strongly correlates with the MR-proADM immunoluminometric sandwich assay (KRYPTOR). 

To our knowledge, this combination of biomarkers has not been analyzed previously in other malignant conditions. It would be reasonable to expect the biomarkers to predict outcome in other types of cancer as well, especially if there is a strong correlation with smoking, such as cancer of the esophagus or head and neck, and subsequently, a high risk of CVD. However, before such conclusions can be drawn, further prospective studies are needed to confirm the findings and enhance our understandings of ST2 and ADM in malignancy.

## 5. Conclusions

In summary, our results show that high levels of MR-proADM and ST2 strongly correlate with a worse patient outcome. These data suggest their potential use in clinical risk stratification and monitoring of SCLC. Although it is unclear to what extent the respective biomarkers contribute to malignant disease, we propose that the predictive values of ADM and ST2 rely on their ability to reveal the subclinical vulnerability of the CV system prior to detectable changes in routine clinical examinations. Future studies are warranted to further elucidate the role of ADM and ST2 as well as other candidate CV biomarkers as guidance for cardiotoxic anti-tumoral therapy in patients with SCLC, perhaps even more importantly with regard to the emergence of immunotherapy. 

## Figures and Tables

**Table 1:** Baseline characteristics and treatment summary of the study population.

	Limited Disease *N* = 104	Extensive Disease *N* = 148
Age, years		
Mean ± SD	67 ± 7.9	66 ± 8.6
Gender, *N* (%)		
Female	61 (59)	85 (57)
Male	43 (41)	63 (43)
Performance status, *N* (%)		
0–1	88 (85)	92 (62)
2–3	16 (15)	56 (38)
Study arm		
LMWH	50 (48)	74 (50)
Control	54 (52)	74 (50)
Biochemistry, median (IQR)		
Hemoglobin, g/L	135 (123–143)	133 (121–142)
Leukocyte count, ×10^9^/L	9.1 (7.2–12.3)	10.2 (7.3–12.8)
Platelet count, ×10^9^/L	314 (261–412)	325 (265–443)
Sodium, mmol/L	139 (135–141)	138 (135–140)
Potassium, mmol/L	4.1 (3.9–4.5)	4.2 (4.0–4.5)
Serum creatinine, µmol/L	65 (57–74)	64 (54–80)
aPTT, s	32 (30–36)	32 (28–35)
Chemotherapy cycles, *N* (%)		
<4 cycles	14 (13)	26 (18)
≥4 cycles	90 (87)	122 (82)
Additional chemotherapy, *N* (%)		
Second line	31 (30)	55 (37)
Third line	9 (9)	6 (4)
No additional chemotherapy	73 (70)	93 (63)
Radiotherapy, *N* (%) ^a^		
Prophylactic cranial	71 (68)	44 (30)
Thoracic	71 (68)	41 (28)
Metastatic lesion	15 (14)	46 (31)
No radiotherapy	11 (11)	41 (30)
Missing	5	12
VTE events, *N* (%)	10 (10)	5 (3)

**^a^** Patients may have received radiotherapy towards more than one site; SD = standard deviation; LMWH = low molecular weight heparin; IQR = interquartile range; aPTT = activated partial thromboplastin time; VTE = venous thromboembolism.

**Table 2:** Unadjusted effects of circulating biomarker levels on overall survival for all patients and by disease stage.

	Quartile 1		Quartile 2		Quartile 3		Quartile 4	
All Patients	HR (95% CI)	*p*-Value	HR (95% CI)	*p*-Value	HR (95% CI)	*p*-Value	HR (95% CI)	*p*-Value
MR-proADM	1.00 (ref.)	<0.001	1.07 (0.73–1.56)	0.730	1.59 (1.08–2.33)	0.018	2.35 (1.61–3.43)	<0.001
MR-proANP	1.00 (ref.)	0.090	1.06 (0.73–1.55)	0.753	1.32 (0.91–1.92)	0.142	1.54 (1.06–2.24)	0.024
Copeptin	1.00 (ref.)	0.007	1.48 (0.95–2.31)	0.083	1.42 (0.91–2.23)	0.124	2.17 (1.40–3.35)	0.001
ADM	1.00 (ref.)	0.001	1.19 (0.80–1.76)	0.384	1.61 (1.09–2.36)	0.016	2.14 (1.46–3.15)	<0.001
ST2	1.00 (ref.)	<0.001	1.34 (0.90–2.02)	0.153	2.23 (1.51–3.29)	<0.001	3.16 (2.14–4.66)	<0.001
Limited disease								
MR-proADM	1.00 (ref.)	0.089	0.78 (0.42–1.43)	0.416	1.58 (0.87–2.89)	0.135	1.50 (0.77–2.90)	0.231
MR-proANP	1.00 (ref.)	0.425	0.72 (0.38–1.37)	0.312	1.04 (0.56–1.94)	0.898	1.25 (0.70–2.24)	0.444
Copeptin	1.00 (ref.)	0.052	1.58 (0.75–3.32)	0.226	0.84 (0.37–1.91)	0.677	2.24 (1.06–4.77)	0.036
ADM	1.00 (ref.)	0.339	0.98 (0.52–1.83)	0.939	1.46 (0.76–2.79)	0.253	1.57 (0.83–2.98)	0.169
ST2	1.00 (ref.)	0.028	0.92 (0.52–1.64)	0.777	1.61 (0.87–2.96)	0.127	2.57 (1.23–5.40)	0.013
Extensive disease								
MR-proADM	1.00 (ref.)	<0.001	1.51 (0.93–2.46)	0.099	1.65 (1.00–2.72)	0.051	3.24 (1.99–5.26)	<0.001
MR-proANP	1.00 (ref.)	0.050	1.28 (0.79–2.07)	0.322	1.41 (0.88–2.27)	0.156	1.99 (1.22–3.26)	0.006
Copeptin	1.00 (ref.)	0.009	1.66 (0.95–2.91)	0.077	2.23 (1.29–3.83)	0.004	2.34 (1.36–4.01)	0.002
ADM	1.00 (ref.)	<0.001	1.55 (0.94–2.58)	0.088	1.70 (1.04–2.75)	0.033	3.03 (1.85–4.95)	<0.001
ST2	1.00 (ref.)	0.001	2.24 (1.25–4.02)	0.007	2.51 (1.46–4.31)	0.001	2.99 (1.78–5.03)	<0.001

HR = hazard ratio; CI = confidence interval; MR-proADM = midregional pro-adrenomedullin; MR-proANP = midregional pro-atrial natriuretic peptide; ADM = adrenomedullin; ST2 = suppression of tumorigenicity 2.

**Table 3:** Adjusted effects of circulating biomarker levels on overall survival for all patients and by disease stage.

	Quartile 1		Quartile 2		Quartile 3		Quartile 4	
All Patients ^a^	Adj HR (95% CI)	*p*-Value	Adj HR (95% CI)	*p*-Value	Adj HR (95% CI)	*p*-Value	Adj HR (95% CI)	*p*-Value
MR-proADM	1.00 (ref.)	0.007	1.12 (0.74–1.69)	0.603	1.39 (0.89–2.16)	0.144	2.18 (1.35–3.51)	0.001
MR-proANP	1.00 (ref.)	0.295	0.83 (0.53–1.29)	0.403	0.91 (0.59–1.41)	0.680	1.23 (0.76–2.00)	0.392
Copeptin	1.00 (ref.)	0.036	1.64 (1.00–2.69)	0.049	1.53 (0.92–2.56)	0.103	2.20 (1.28–3.78)	0.004
ADM	1.00 (ref.)	0.041	1.24 (0.81–1.90)	0.317	1.41 (0.91–2.18)	0.126	2.00 (1.23–3.24)	0.005
ST2	1.00 (ref.)	0.004	1.47 (0.93–2.33)	0.101	2.19 (1.37–3.52)	0.001	2.40 (1.44–3.98)	0.001
Limited disease ^b^								
MR-proADM	1.00 (ref.)	0.405	0.69 (0.36–1.32)	0.261	1.27 (0.65–2.48)	0.487	1.01 (0.46–2.24)	0.974
MR-proANP	1.00 (ref.)	0.160	0.42 (0.20–0.90)	0.025	0.70 (0.35–1.40)	0.312	0.54 (0.23–1.26)	0.153
Copeptin	1.00 (ref.)	0.331	1.56 (0.65–3.77)	0.320	0.85 (0.33–2.19)	0.728	1.69 (0.62–4.62)	0.308
ADM	1.00 (ref.)	0.521	0.79 (0.40–1.54)	0.486	1.34 (0.67–2.69)	0.403	0.95 (0.42–2.13)	0.893
ST2	1.00 (ref.)	0.128	0.86 (0.45–1.64)	0.641	1.45 (0.70–3.00)	0.318	2.39 (0.96–5.98)	0.063
Extensive disease ^b^								
MR-proADM	1.00 (ref.)	0.001	1.59 (0.89–2.84)	0.115	1.57 (0.86–2.89)	0.145	3.49 (1.84–6.60)	<0.001
MR-proANP	1.00 (ref.)	0.313	1.16 (0.65–2.05)	0.622	1.09 (0.61–1.93)	0.775	1.69 (0.89–3.21)	0.110
Copeptin	1.00 (ref.)	0.054	1.73 (0.92–3.23)	0.088	2.08 (1.11–3.87)	0.022	2.42 (1.23–4.77)	0.010
ADM	1.00 (ref.)	0.012	1.68 (0.94–2.99)	0.080	1.53 (0.86–2.70)	0.145	2.88 (1.52–5.47)	0.001
ST2	1.00 (ref.)	0.002	3.05 (1.54–6.02)	0.001	3.03 (1.56–5.91)	0.001	3.43 (1.73–6.79)	<0.001

**^a^** Adjusted for disease stage, age, gender, performance status (0–1 vs. 2–3), leukocyte count (≤9.0 vs. >9 × 10^9^/L) and sodium levels (<136 vs. ≥136 mmol/L). **^b^** Adjusted for age, gender, performance status (0–1 vs. 2–3), leukocyte count (≤9.0 vs. >9 × 10^9^/L) and sodium levels (<136 vs. ≥136 mmol/L). Adj HR = adjusted hazard ratio; CI = confidence interval; MR-proADM = midregional pro-adrenomedullin; MR-proANP = midregional pro-atrial natriuretic peptide; ADM = adrenomedullin; ST2 = suppression of tumorigenicity 2.

## Data Availability

The data presented in this study are available from the corresponding author on reasonable request.

## Supplementary Materials

The following supporting information can be downloaded at: https://www.mdpi.com/article/10.3390/cancers14051307/s1, Figure S1: Consort diagram; Table S1: Biomarkers at baseline; Figure S2: Correlations between adrenomedullin levels assessed as MR-proADM (KRYPTOR) and by proximity extension assay (OLINK); Figure S3: Kaplan–Meier analysis of overall survival by ADM (OLINK) levels; Figure S4: Kaplan–Meier analysis of overall survival by MR-proANP levels; Figure S5: Kaplan–Meier analysis of overall survival by copeptin levels; Figure S6: Kaplan-Meier analysis of progression-free survival by combined biomarker score of MR-proADM and ST2.
