# Supplementary material for: Circulating Levels of the Cardiovascular Biomarkers ST2 and Adrenomedullin Predict Outcome within a Randomized Phase III Lung Cancer Trial (RASTEN)
Source: Cancers (Basel). 2022 Mar 3;14(5):1307. doi: 10.3390/cancers14051307 (PMC8909619; doi:10.3390/cancers14051307)
Supplement: Supplementary file 1 [file cancers-14-01307-s001.zip › cancers-1611276-supplementary/Supplementary Figure S3.pdf]

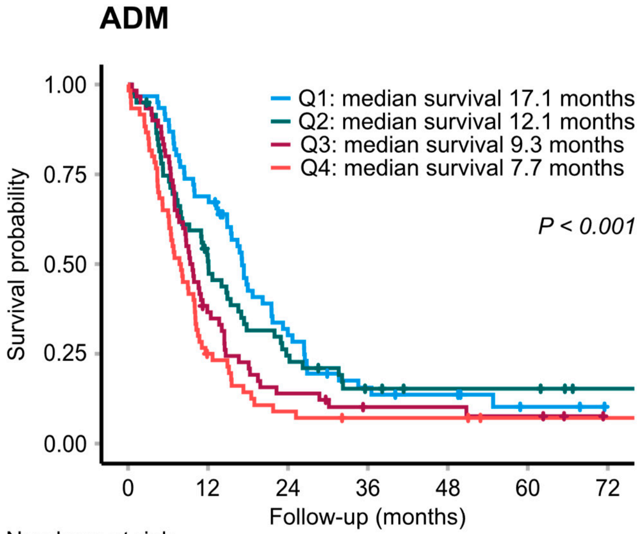

Q1	61	42	17	8	6	2	0
Q2	60	29	14	7	5	5	2
Q3	60	21	8	4	4	3	0
Q4	60	14	5	3	3	1	1

Supplementary Figure S3. Kaplan-Meier analysis of overall survival by ADM (OLINK) levels, all patients.
