# Supplementary material for: Circulating Levels of the Cardiovascular Biomarkers ST2 and Adrenomedullin Predict Outcome within a Randomized Phase III Lung Cancer Trial (RASTEN)
Source: Cancers (Basel). 2022 Mar 3;14(5):1307. doi: 10.3390/cancers14051307 (PMC8909619; doi:10.3390/cancers14051307)
Supplement: Supplementary file 1 [file cancers-14-01307-s001.zip › cancers-1611276-supplementary/Supplementary Figure S5.pdf]

Copeptin

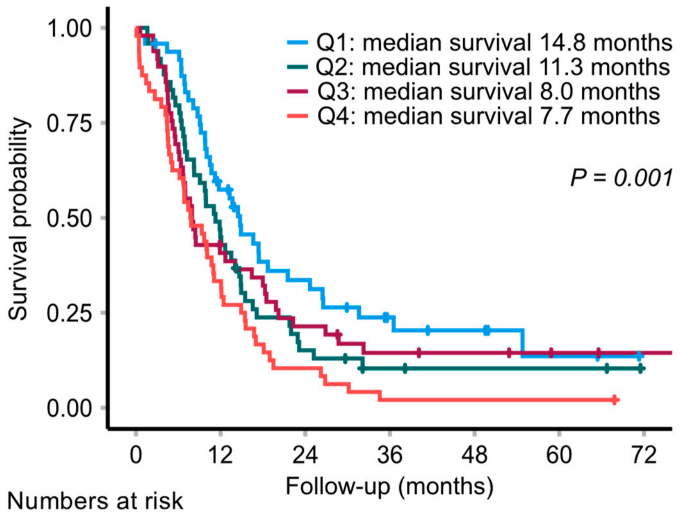

Numbers at risk

Q1	48	26	14	7	5	2	0
Q2	49	22	7	3	2	2	0
Q3	49	19	10	6	5	3	2
Q4	48	16	5	1	1	1	0

Supplementary Figure S5. Kaplan-Meier analysis of overall survival by copeptin levels, all patients.
