# Supplementary material for: Circulating Levels of the Cardiovascular Biomarkers ST2 and Adrenomedullin Predict Outcome within a Randomized Phase III Lung Cancer Trial (RASTEN)
Source: Cancers (Basel). 2022 Mar 3;14(5):1307. doi: 10.3390/cancers14051307 (PMC8909619; doi:10.3390/cancers14051307)
Supplement: Supplementary file 1 [file cancers-14-01307-s001.zip › cancers-1611276-supplementary/Supplementary Table S1_revised.pdf]

Supplementary Table S1. Biomarkers at baseline, for all patients and by disease extent.

**Vasoactive Peptide**	**All Patients**		**Limited Disease**		**Extensive Disease**	
	**Median (IQR)**	***N***	**Median (IQR)**	***N* (%)**	**Median (IQR)**	***N* (%)**
MR-proADM, nmol/L	0.76 (0.64-0.94)	252	0.71 (0.61-0.89)	104 (41)	0.79 (0.65-1.03)	148 (59)
MR-proANP, pmol/L	75.6 (48.8-112.0)	251	75.0 (45.9-116.9)	104 (41)	75.8 (52.2-106.8)	147 (59)
Copeptin, pmol/L	7.7 (3.8-20.7)	194	6.9 (3.8-17.6)	78 (40)	8.5 (3.9-32.1)	116 (60)
ADM, a.u.	7.3 (7.0-7.7)	241	7.3 (7.0-7.7)	99 (41)	7.4 (7.0-7.7)	142 (59)
ST2, a.u.	4.8 (4.2-5.9)	241	4.6 (3.9-5.1)	99 (41)	5.3 (4.5-6.5)	142 (59)

IQR = Interquartile range; MR-proADM = Midregional pro-adrenomedullin; MR-proANP = Midregional pro-atrial natriuretic peptide; ADM = adrenomedullin; a.u. = Arbitrary unit; ST2 = Suppression of tumorigenicity 2.
